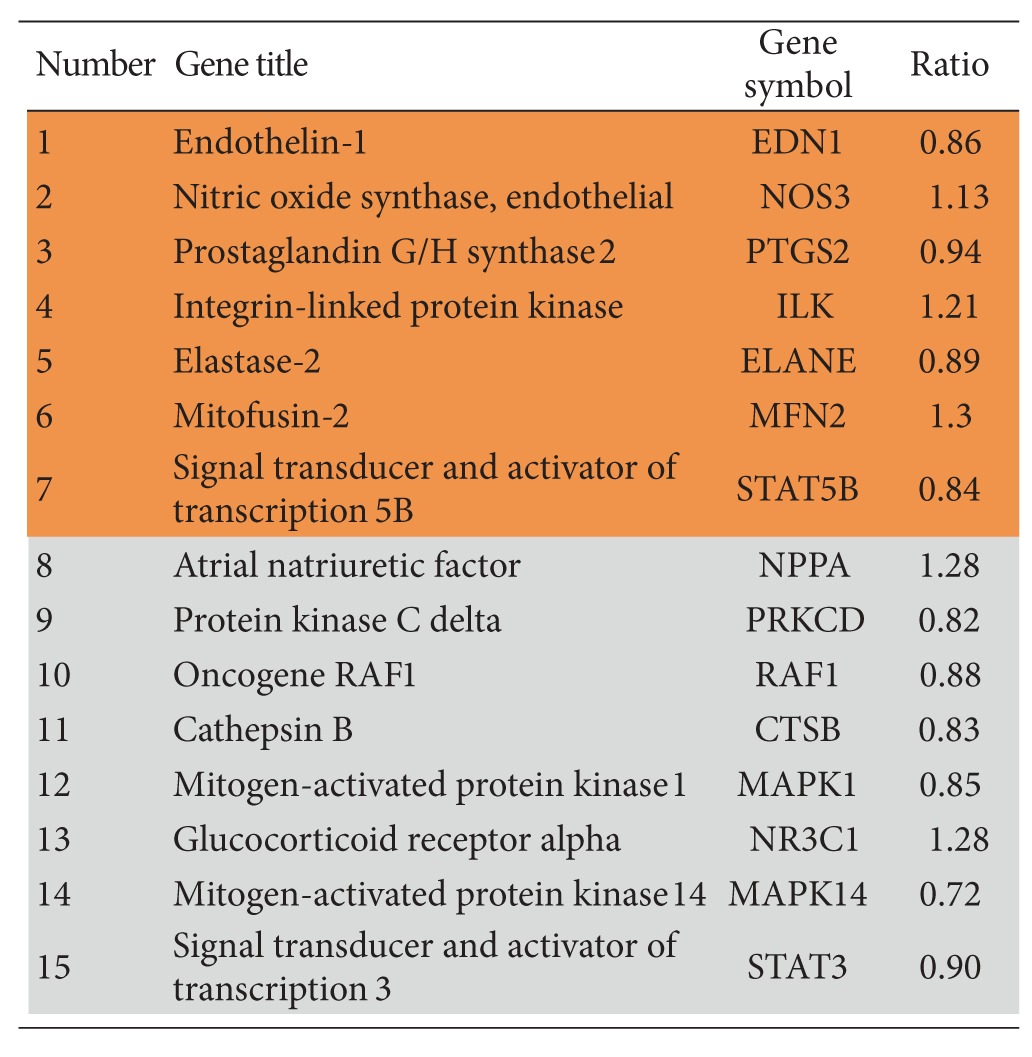# Gene Expression Profiling on the Molecular Action of Danshen-Gegen Formula in a Randomized Placebo-Controlled Trial of Postmenopausal Women with Hypercholesterolemia

**DOI:** 10.1155/2013/703705

**Published:** 2013-09-23

**Authors:** Chi-Man Koon, Chun-Hay Ko, Xu-Xu Sun, Sandy Wan-Heng Hoi, Jacqueline Chor-Wing Tam, David Wing-Shing Cheung, King-Fai Cheng, Suet-Yee Pang, Wing-Man Lo, Ping Chook, Clara Bik-San Lau, Wai-Yee Chan, Ping-Chung Leung, Timothy Chi-Yui Kwok, Kwok-Pui Fung

**Affiliations:** ^1^Institute of Chinese Medicine, The Chinese University of Hong Kong, Shatin, New Territories, Hong Kong; ^2^State Key Laboratory of Phytochemistry & Plant Resources in West China, The Chinese University of Hong Kong, Shatin, New Territories, Hong Kong; ^3^School of Biomedical Sciences, The Chinese University of Hong Kong, Shatin, New Territories, Hong Kong; ^4^Department of Medicine and Therapeutics, The Chinese University of Hong Kong, Shatin, New Territories, Hong Kong; ^5^The Chinese University of Hong Kong-Zhejiang University Joint Laboratory on Natural Products and Toxicology Research, Shatin, New Territories, Hong Kong

## Abstract

The Danshen-Gegen formula (DG) is a traditional Chinese herbal formula which has long been used to treat cardiovascular disease. DG was found to be a cardiovascular tonic in our recent research. However, a comprehensive investigation of the molecular mechanism of DG in cardiovascular disease has not been performed. The aim of this study was to clarify the transcriptional profiling of genes modulated by DG on postmenopausal women by using DNAmicroarray technology. We obtained 29 whole blood samples both from DG-treated and placebo-treated subjects. Blood lipid profile and intima-media thickness (IMT) were measured. Affymetrix GeneChip was used to identify differentially expressed genes (DEGs), followed by validation by the real-time PCR method. The results showed that DG-treated group has a significant improvement in IMT and lipid profile as compared to placebo-treated group. For the genomic study, the DG-treated group has a higher number of DEGs identified as compared to the placebo-treated group. Two important biological processes of “regulation of systemic arterial blood pressure by hormone” and “regulation of smooth muscle proliferation” have been identified by GePS in the DG-treated group. No significant biological process and cellular components were identified in the placebo-treated group. This genomic study on the molecular action of DG in postmenopausal women gathered sufficient molecular targets and pathways to reveal that DG could improve neointima thickening and hypertension.

## 1. Introduction

The term “postmenopause” is applied to women who have not experienced menstruation for a minimum of 12 months. The increasing incidence of cardiovascular disease in postmenopausal women could be related to estrogen deprivation which has unfavorable effects on blood vessels, lipid profile, and blood pressure [1]. The most common postmenopause treatment is hormone replacement therapy (HRT). HRT introduces synthetic hormones into a woman's body and helps to eliminate postmenopausal symptoms. However, HRT has many side-effects, such as heart disease, blood clots, and breast cancer, ovarian cancer [2]. Besides medical therapy, another option to help postmenopausal women to alleviate their symptoms is alternative medicine.

Traditional Chinese medicine (TCM) is one of the alternative medicines which originated in ancient China and which has evolved over thousands of years ago. TCM is not only commonly used in Asian countries but also in America because it has fewer side effects as compared to prescription medicines. Prescription medicine always has a specific action with strong efficiency in treating a specific syndrome, but it can produce side-effects if there is long-term usage. However, most diseases, especially the chronic diseases such as cardiovascular diseases (CVDs), are multifactorial. CVDs have many risk factors, such as hyperlipidemia, hypertension, and vascular thickening (e.g., IMT) in which IMT is used to track the regression, arrest, or progression of atherosclerosis. Patients very often need to take a series of medications. TCM always comes in a combination of herbs with various chemical components that may act in an additive or synergistic fashion [3]. The biological action of the multiple chemical components of TCM may show multitargeting effects and act through multiple molecular pathways in order to achieve a balance of body functions.

Danshen and Gegen formula (DG) is a modified herbal formula used in traditional Chinese medicine; it consists of two herbs which exhibited prominent effects on hypertension [4–6], atherosclerosis [3, 7, 8], and cardioprotection [9–11] in our laboratory studies. In our previous clinical trial, this formula improved the cardiovascular functions in patients, such as producing a mild decrease in low-density lipoprotein (LDL), improved brachial flow-mediated dilation (FMD), and IMT, such patients having severe cardiovascular problems [12]. The current clinical study aimed at using DG to diminish the process of cardiovascular deterioration in postmenopausal women by measuring the IMT, lipid profile, and blood pressure. Secondly, this genomic study employed the use of whole blood samples to serve as a platform and it aimed to correlate the molecular action of DG with clinical outcomes by using the DNA microarray technology. Furthermore, the application of microarray technology and associated bioinformatic data mining tools could simultaneously allow the analysis of a large number of molecular targets which are possibly associated with the biological effects of DG.

## 2. Materials and Methods

### 2.1. Herbal Preparation and Identification of Chemical Markers

The procedures used to make the herbal preparation and the identification of chemical markers were the same as in our previous report [5]. In brief, the raw materials were washed to remove all trace particles and contaminants. The raw components were then cut into a uniform size and mixed in the weight ratio of Danshen to Gegen of 7 : 3 (DGW). The raw mixture was allowed to soak with 10-fold of water (v/w) for 1.5 hours followed by extraction at 100°C for 1 hour. The residue was then subjected to two more subsequent extractions for 1 hour and then for half an hour. The combined extracts were then dried at −660 mmHg and 60°C and stored in desiccators at room temperature for future use. The dried herbal extract was dissolved in a culture medium and sterilized by filtration before being added to the cells. The liquid chromatographic system which we used was the HPLC system equipped with a photodiode array (PDA) UV detector. The chromatographic separation of the chemical markers was achieved by using an Agilent Eclipse XDB-C18 column. A gradient elution (1 mL/min) was achieved by using 0.5% acetic acid in acetonitrile (solvent A) and 0.5% acetic acid in water (solvent B) as the mobile phases.

### 2.2. Study Design and Populations

All subjects were 45–65-year-old postmenopausal women whose menstruation had ceased more than 12 months previously and whose fasting serum LDL was ≧3.5 mmol/L and <4.9 mmol/L. None of the subjects were taking hormones, statins, and nutritional supplements. Furthermore, women who have had children and who have a normal diet (nonvegetarian) were eligible. The detailed inclusion and exclusion criteria of this clinical trial are listed in the supplementary information available on line at http://dx.doi.org/10.1155/2013/703705.

Eligible subjects were screened at the CUHK Chinese Medicine Research Centre. After signing written informed consents, they were clinically assessed. Then the subjects were randomized to take two capsules of DG (1 gram), or two capsules of an image-identical placebo (1 gram) daily, in double-blind and parallel fashion for 12 months. The placebo capsule contained medical starch and a natural sugar-processed pigment in a 2 : 1 ratio. Peripheral blood samples were collected from the subjects before and after DG or placebo treatment. Clinical visits for progress monitoring were arranged at 12-week intervals. Blood tests and ultrasound vascular assessments were repeated at 52 weeks. Ultrasound vascular assessment was performed by using B-mode ultrasonography: carotid artery scan (7–10 MHz probe, color Doppler). Centralized measurement of IMT of the common carotid artery was performed by using verified automatic measurement IMT software. The primary objective was to evaluate the efficacy of DG treatment for preventing atherosclerosis in postmenopausal women who have borderline hypercholesterolemia.

### 2.3. Ethics Approval

 Prior to study commencement, the study protocol, the informed consent documents, and any other appropriate documents were submitted to the Clinical Research Ethics Committee (CREC), Joint The Chinese University of Hong Kong—New Territories East Cluster. CREC approval number is CRE-2008.342-T. The participants in the study had given written informed consent. Approval was granted on October 21, 2008.

### 2.4. Blood Collection, RNA Purification, RNA Quantification, and RNA Integrity

 Three subjects from the DG group and three subjects from the placebo treatment group were selected for the microarray study. Whole blood samples were collected in PAXgene blood RNA tubes (Qiagen, USA) which contain a proprietary reagent composition to protect RNA molecules from degradation by RNases and to minimize *ex vivo* changes in the gene expression. The samples were gently inverted and stored at −80°C within two hours of collection.

 RNA was extracted from whole blood by using the PAXgene Blood RNA Kit in accordance with the manufacturer's guidelines. Briefly, the samples were removed from −80°C and incubated at room temperature for two hours to ensure complete lysis. Following cell lysis, nucleic acids were pelleted and treated with a buffer containing proteinase-K. After digestion with RNase-free DNase (Qiagen), the RNA was subsequently purified on PAXgene spin columns and eluted in 40 *μ*L of elution buffer. 

 Freshly extracted RNA was measured by using a NanoDrop 2000 UV-visible spectrophotometer (Thermo Scientific, USA). RNA integrity was additionally assessed by using a Eukaryote total RNA nanochip and the Agilent 2100 Bioanalyser (Agilent Technologies, Germany). 

### 2.5. Microarray Hybridization

cRNA was transcribed *in vitro* by incorporating a biotinylated pseudouridine molecule by using GeneChip Expression 3′-Amplification Reagents for IVT Labeling, over 16 hours at 37°C. Hybridization was performed by using GeneChip Human Gene 1.0 ST Array (Affymetrix) containing 764,885 distinct probes corresponding to 28,869 well-annotated genes. After washing, the chips were stained with streptavidin-phycoerythrin in accordance with the Affymetrix EukGE-WS2v5 protocol by using a Fluidic FS450 station. The microarrays were read with the GeneChip Scanner 3000 (Affymetrix). The Affymetrix GeneChip Operating Software version 1.2 was used to manage the Affymetrix GeneChip array data and to automate the control of the GeneChip fluidics stations and scanners.

### 2.6. Analyses for Genomic Study

The acquisition and initial quantification of array images were conducted using the AGCC software (Affymetrix). The subsequent data analyses were performed by using Partek Genomics Suite Version 6.4 (Partek, USA). We first performed a one-way ANOVA to identify the genes between the groups at a *P* value of less than 0.05 and then calculated the relative difference in fold change (FC) between the groups. Although these criteria were used with low stringency, this is expected to detect more genes truly associated with DG treatment at the expense of increasing the number of false positives to be validated by qPCR bioinformatics and experimental means. Cluster analyses and principal component analysis (PCA) were conducted with Partek default settings. Comprehensive data and literature mining was performed through Genomatix Pathway System (GePS) (http://www.genomatix.de/) for extracting gene relationships based on information extracted from public proprietary databases.

### 2.7. Quantitative Real-Time PCR Analysis

Quantitative Real-Time PCR (qPCR) confirmation of the selected genes was performed by using iScript one-step RT-PCR Kit with SYBR Green (Bio-rad) on CFX96 Real-Time PCR Detection System (Bio-Rad) in accordance with the manufacturer's instructions. The RNA from the remaining subjects in the treatment group (*n* = 18) and the placebo group (*n* = 11) was used for qPCR confirmation of the selected genes. The primer list for qPCR is shown in Table 1. The threshold cycle (Ct), the cycle number at which the amount of amplified genes of interest reached a fixed threshold, was determined. Relative expression of the RT-PCR product was calculated by using the comparative 2^−ΔΔCt^ method. The endogenous control GAPDH was used for normalization. The fold difference of posttreatment was then determined by normalizing all values to the mean of the relative expression for pretreatment. The differences in the gene expression level between the DG-treated and the placebo-treated group were compared by a student *t*-test; a probability of *P* < 0.05 was considered to be statistically significant. 

### 2.8. Statistical Analyses

Baseline characteristics are presented as descriptive statistics (frequency (%) or mean, standard deviation (SD), and standard error (SE) minimum, median, and maximum). The data were processed to give group mean values and standard error. Differences within a group were compared by a paired *t*-test. Differences between groups were compared by the Mann-Whitney test. Significance level was defined as *α* = 0.05. Data were analyzed by using the SPSS software (version 16.00, SPSS Inc.).

## 3. Results

### 3.1. Patient Characteristics

There were no differences detected in any patient baseline characteristics between the DG-treated group and the placebo-treated group included in the primary outcome IMT (Table 2).

### 3.2. Effect of DG on Carotid IMT

**Figure 1:**
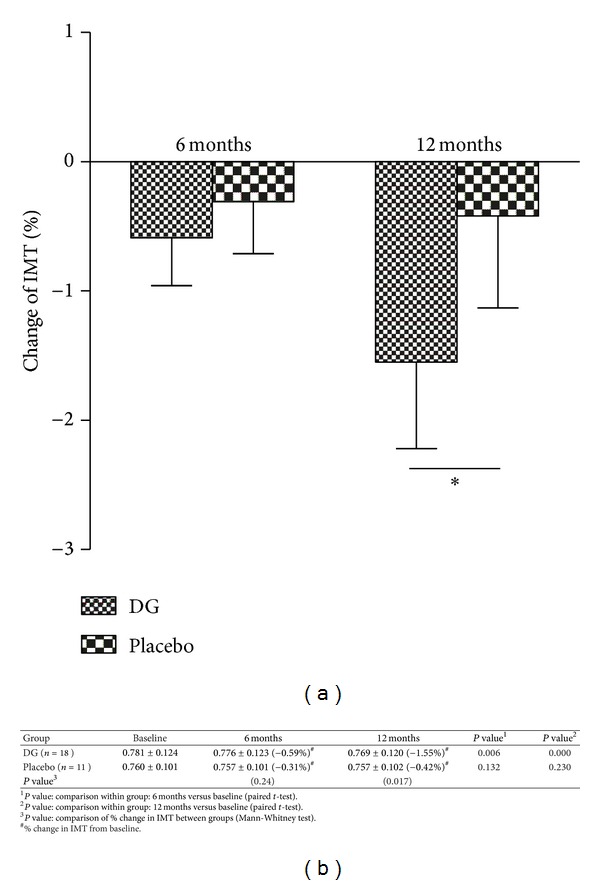
(a) Percentage changes from baseline in IMT at 6-month and 12-month DG or placebo treatment, *P < 0.05; (b) IMT of subjects at baseline, 6-month, and 12-month DG or placebo treatment (in mm).

When compared to the baseline, there was a significant improvement in IMT in the DG group after six months (*P* = 0.006) and after 12 months (*P* < 0.001). However, no such significant change was observed in the placebo group (Figure 1(b)). In the DG group, IMT was significantly improved 1.55% after 12-month treatment as compared to only 0.42% in the placebo group (*P* = 0.017), as shown in Figure 1(a). 

### 3.3. Effect of DG on Blood Pressure

At the end of the study, the blood pressure of all the subjects both in the DG-treated and the placebo-treated group was within normal range. There were no statistical differences of systolic and diastolic blood pressure between the two groups for the baseline measurement and during the study period (Table 3).

### 3.4. Effect of DG on Blood Lipid Profile

**Figure 2:**
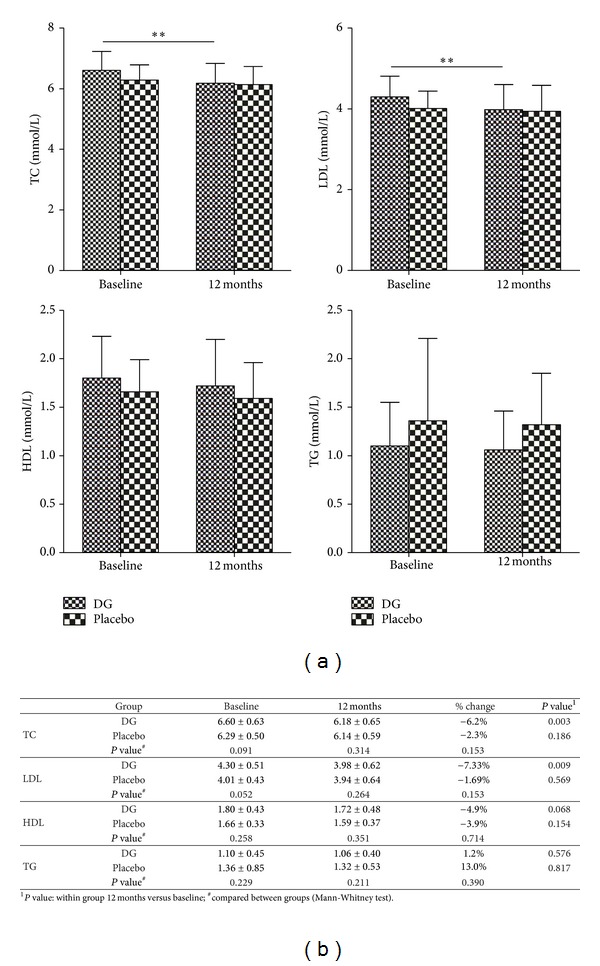
(a) TC, LDL, HDL, and TG in mmol/L at 12 months compared with baseline, **P < 0.01; (b) percentage change in blood lipid profile (TC, LDL, HDL, and TG) of subjects after 12-month DG or placebo treatment.

Blood lipid levels are shown in Figure 2(a). After 12-month treatment, a remarkable decrease in total cholesterol (TC) and low-density lipoprotein (LDL) in the DG-treated group was observed but not in the placebo-treated group. In the DG-treated group, TC and LDL were significantly decreased (6.2% and 7.3%) when compared to the corresponding baseline (*P* = 0.003 and *P* = 0.009). No such change was observed in the placebo-treated group (*P* = 0.186 and *P* = 0.569). However, the percentage change of TC and LDL in the DG-treated group was not significantly different from that in the placebo-treated group (*P* = 0.153) (Figure 2(b)). However, there was no significant change in high-density lipoprotein (HDL) and triglyceride (TG) in both groups after 12-month treatment (Figure 2(b)).

### 3.5. Differential Expressed Genes (DEGs)

**Figure 3:**
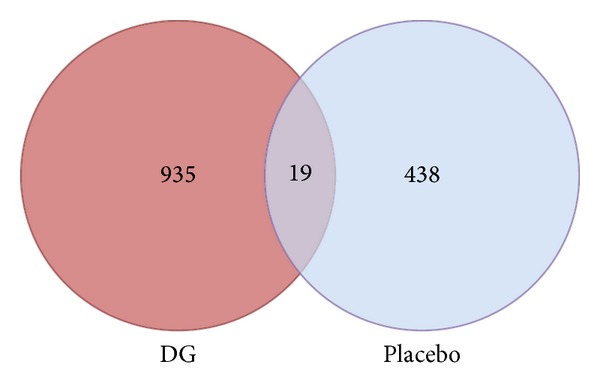
Venn diagram of differential expressed genes (DEGs) in the DG-treated group and the placebo-treated group. Nonoverlapped area is the number of DEGs found only in corresponding group. Overlapped area is the number of common DEGs shared between the DG-treated group (*n* = 3) and the placebo group (*n* = 3).

The differential gene expression of two whole blood samples (Pre: before treatment; Post: after 12 months of treatment) was compared in the DG-treated group (*n* = 3) and the placebo-treated group (*n* = 3)  (*P* < 0.05). For the DG-treated group, there were 954 differentially expressed genes (DEGs) with 485 being upregulated and 469 being downregulated. For the placebo group, there were 457 DEGs with 290 upregulated and 167 downregulated. There were only 19 common DEGs shared between the DG-treated group and the placebo-treated group, as shown in the Venn diagram (Figure 3). 

### 3.6. Principal Component Analyses (PCA) and Cluster Analyses

**Figure 4:**
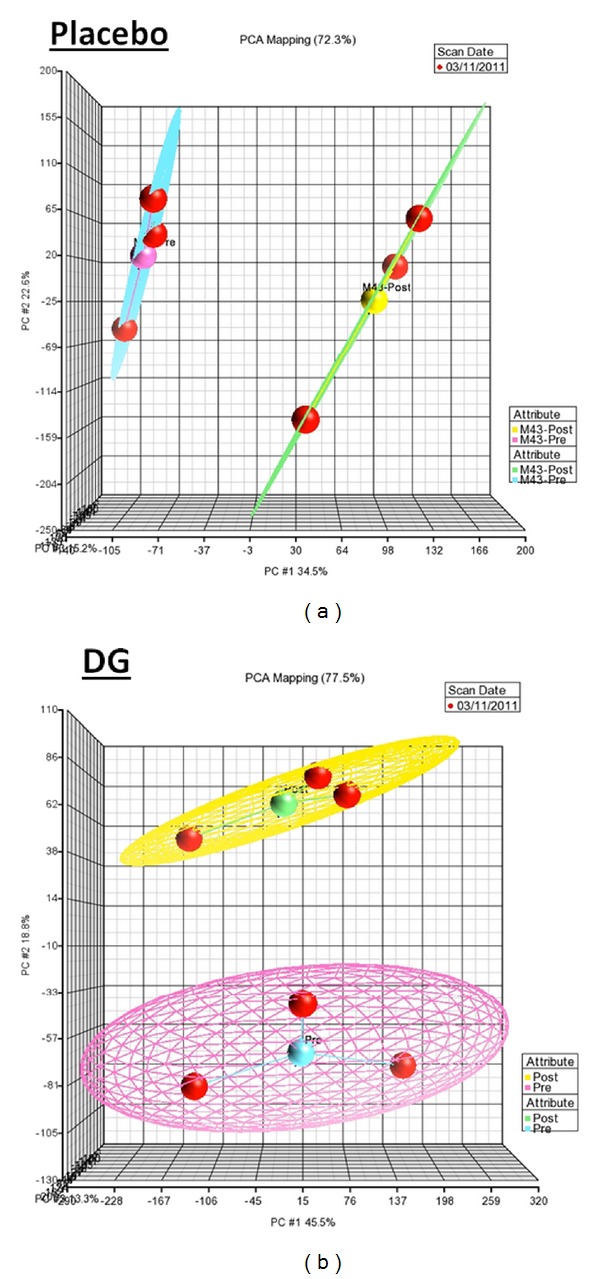
PCA analysis of Pre and Post samples of the DG-treated group and the placebo-treated group. Each Pre and Post sample of each group was repeated in three individual hybridizations. The cluster of three red balls shows the intraexperimental variation of each sample.

**Figure 5:**
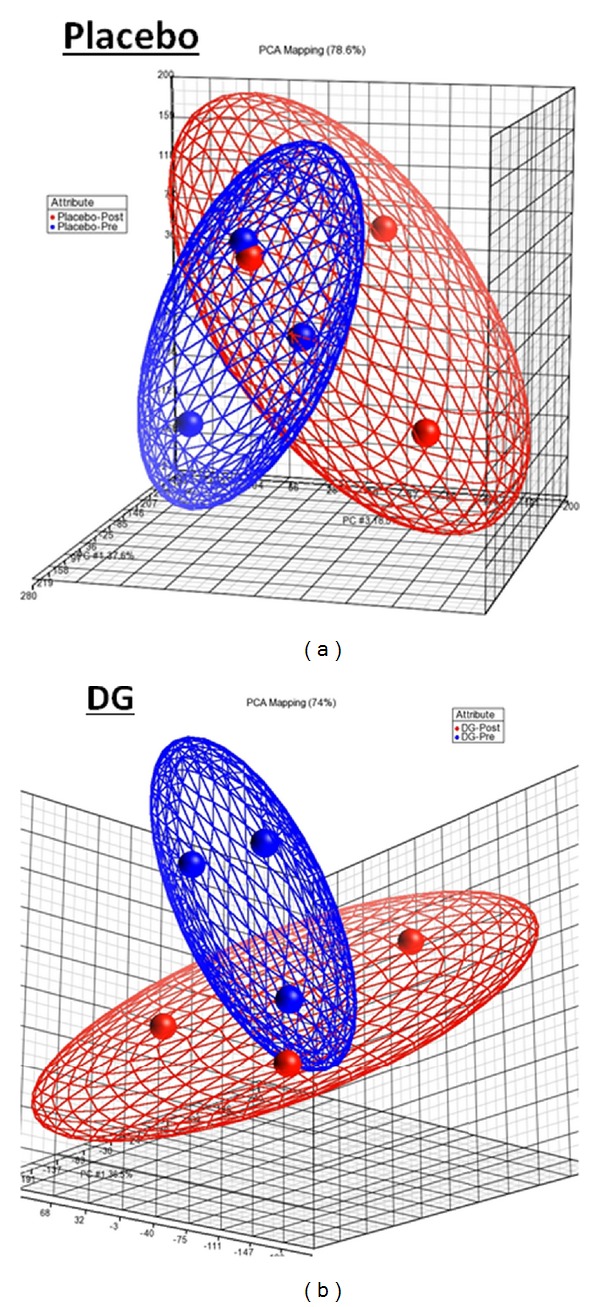
Cluster analyses of Pre and Post samples in three individual subjects of the DG-treated group and the placebo-treated group. The overlapped area of the clusters (red and blue) indicates the greater similarity of the gene expression profile.

To evaluate intraexperimental technical variation, the gene expression profile of three repeated hybridizations of each sample was performed by using principal component analysis (PCA). The PCA results revealed that there was no overlapping in the clusters of three repeated hybridizations (red balls) of the Pre and Post samples of the DG-treated group and the placebo-treated group (Figure 4). The cluster analyses of Pre and Post whole blood samples of the DG-treated group and the placebo-treated group (*n* = 3) are shown in Figure 5. The gene expression profile (as shown in cluster) of the Pre samples was well separated from that of the Post samples in the DG-treated group, whereas the cluster of the Pre sample was overlapped with that of the Post samples in the placebo-treated group (Figure 5). 

### 3.7. Data and Literature Mining by Genomatix Pathway System (GePS)

**Figure 6:**
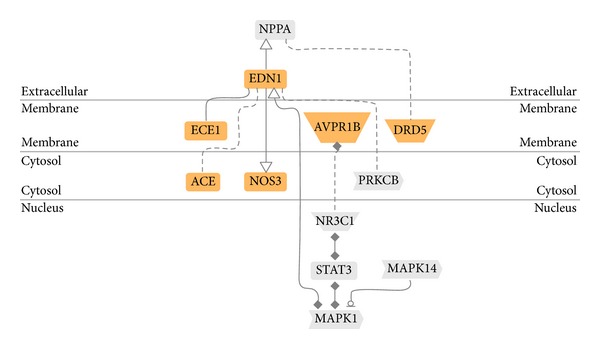
A pathway of “regulation of systemic blood pressure by hormone” was generated by the GePS program from Genomatix that connects the DEGs found after DG treatment for 12 months.

**Figure 7:**
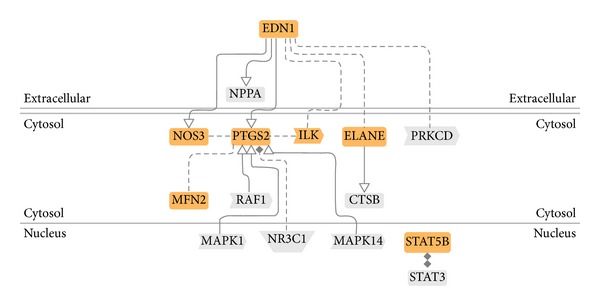
A pathway of “regulation of smooth muscle cell proliferation” was generated by the GePS program from Genomatix that connects the DEGs found after DG treatment for 12 months.

The biological processes were presented by an AmiGo gene ontology (GO) database via the Genomatix Pathway System (GePS) for extracting DEGs relationships based on information extracted from public proprietary databases. The two most important biological processes of “regulation of systemic arterial blood pressure by hormone” and “regulation of smooth muscle proliferation” were identified by GePS in the DG-treated group. No significant biological process and cellular components were identified in the placebo-treated group. For the biological process of “regulation of systemic arterial blood pressure by hormone,” six genes were identified with a *P* value of 5.98 × 10^−5^. For the biological process of “regulation of smooth muscle proliferation,” seven genes were identified with a *P*-value of 1.79 × 10^−3^. These identified genes are shown in orange color while the co-citation genes are shown in grey color in Figures 6 and 7. 

The most significantly regulated “blood pressure-” related genes included endothelin-1 (EDN1), endothelin-converting enzyme-1 (ECE1), dopamine D5 receptor (DRD5), vasopressin V1b receptor (AVPR1B), angiotensin-converting enzyme (ACE), and endothelial nitric oxide synthase (NOS3) and its co-citation genes atrial natriuretic factors (NPPA), protein kinase C beta type (PRKCB), signal transducers and activators of transcription 3 (STAT3), glucocorticoid receptor alpha 2 (NR3C1), mitogen-activated protein kinase 1 (MAPK1), and mitogen-activated protein kinase 14 (MAPK14) (Table 4). The ratio indicated the upregulation (>1.00) or downregulation (<1.00). There were four upregulated genes and eight downregulated genes in those pathways. The most significantly regulated “smooth muscle proliferation-” related genes included EDN1, NOS3, prostaglandin G/H synthase 2 (PTGS2), integrin-linked protein kinase (ILK), Elastase-2 (ELANE), mitofusin-2 (MFN2) and signal transducers and activators of transcription 5B (STAT5B) and its co-citation genes NPPA, protein kinase C delta (PRKCD), RAF1, cathepsin B (CTSB), MAPK1, NR3C1, mitogen-activated protein kinase 14 (MAPK14), and STAT3 (Table 5). There were five upregluated genes and ten downregulated genes in those pathways.

**Figure 8:**
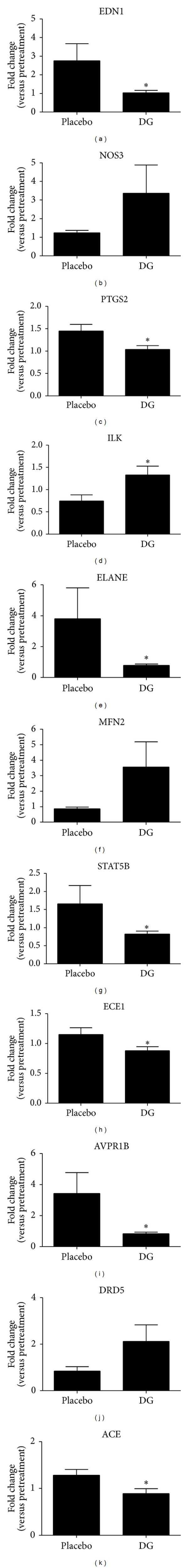
Gene expression level of the 11 DEGs after DG and placebo treatment. The RNA from the remaining subjects in the treatment group (*n* = 18) and the placebo group (*n* = 11) was used. The *y*-axis represents fold change (posttreatment versus pretreatment). The bar on the column stands for the mean ± S.E.M., *P < 0.05 as compared to the placebo group.

 The gene expression level of the 11 DEGs, both in the “blood pressure” and “smooth muscle proliferation” pathways, is shown in Figure 8. The DG-treated group has a significant lower fold change in EDN1 (2.76 versus 1.04), PTGS2 (1.45 versus 1.04), ELANE (3.81 versus 0.80), STAT5B (1.66 versus 0.82), ECE1 (1.15 versus 0.88), AVPR1B (3.43 versus 0.83), and ACE (1.28 versus 0.89) gene expression as compared to the placebo-treated group. The DG-treated group has a significant higher fold change in NOS3 (1.24 versus 3.36), ILK (0.74 versus 1.33), MFN2 (0.86 versus 3.56), and DRD5 (0.85 versus 2.12) gene expression as compared to the placebo-treated group. 

**Figure 9:**
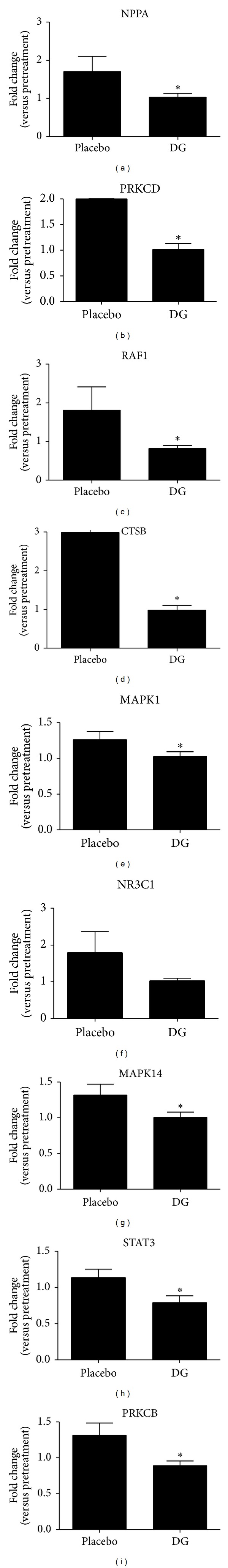
qPCR analyses of the nine co-citation genes after DG and placebo treatment. The RNA from the remaining subjects in the treatment group (*n* = 18) and the placebo group (*n* = 11) was used. The *y*-axis represents fold change (posttreatment versus pretreatment). The bar on the column stands for the mean ± S.E.M., *P < 0.05 as compared to the placebo group.

The gene expression level of the nine co-citation genes both in the “blood pressure” and “smooth muscle proliferation” pathways is shown in Figure 9. The DG-treated group has a significant lower fold change in all co-citation genes as compared to the placebo-treated group, NPPA (1.71 versus 1.03), PRKCD (2.08 versus 1.02), RAF1 (1.81 versus 0.82), CTSB (2.99 versus 0.99), MAPK1 (1.26 versus 1.03), NR3C1 (1.80 versus 1.03), MAPK14 (1.32 versus 1.01), STAT3 (1.14 versus 0.79), and PRKCB (1.31 versus 0.89).

**Figure 10:**
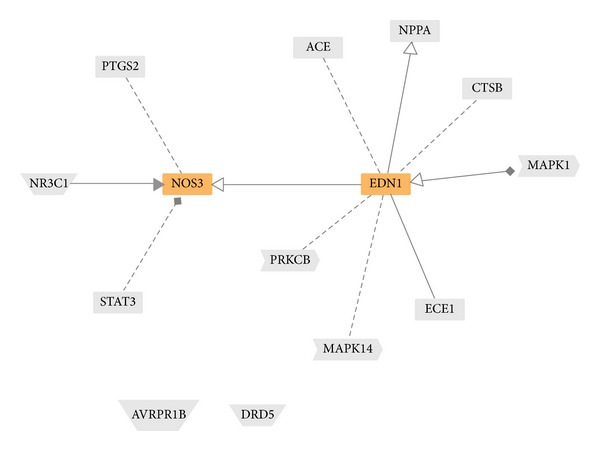
Integration of DEGs in two biological processes (blood pressure and smooth muscle proliferation) by using GePS. Two core DEGs (NOS and EDN1) are identified by highlighting in orange color.

By the integration of DEGs in these two biological processes and by performing network analysis using GePS, which dynamically generates functional association networks based on the curated literature information of protein-protein interaction, coexpression, and genetic regulation, core DEGs can be identified.  Figure 10 shows the network with the basis of those DEGs. Both EDN1 and NOS3 are the core of the network, as highlighted. 

## 4. Discussion

The cellular and biological basis of DG on the cardiovascular tonic effect has been demonstrated in our previous preclinical and clinical studies [3, 5–12]. However, the molecular mechanisms of DG which exert their effects via multiple targets in a complex network are difficult to identify. Many factors, such as ion channels, protein-influencing calcium homeostasis, cytokines, growth factor, metabolic, and inflammatory stressors, may be involved in these molecular mechanisms [13]. With the help of the data and literature mining tool (GePS), it is now possible to extract gene relationships based on information extracted from public proprietary databases and therefore targeted network pathways. The present study using microarray analysis demonstrated that hundreds of differentially expressed genes (DEGs) were modulated in subjects with DG treatment. These findings suggest that these DEGs play critical roles in the pathways of hypertension and the pathophysiology of atherosclerosis (smooth muscle cell proliferation). In addition, the results of the molecular findings may correlate with the clinical outcomes. 

The present clinical study was designed to evaluate the efficacy of DG treatment in the prevention of atherosclerosis in postmenopausal women who have borderline hypercholesterolemia. Postmenopausal women are associated with changes in their metabolism together with new cardiovascular risk factors because of the changes of their hormonal status. The risk factors are the changes of lipid profile, obesity, hypertension, and atherosclerosis [14]. The results found that DG reduced the IMT significantly and that it decreased the TC and LDL. Although there was no change in blood pressure in the DG-treated group, it did not mean DG has no such effect; this is because all the subjects had normal blood pressure during the study period.

For the microarray study, a larger change in gene expression was found in the DG-treated group (Figure 3), and this was correlated with the number of DEGs (the DG-treated group has twofold more DEGs than the placebo-treated group, 954 versus 457). Hence, DG may exert certain effects from the genomic point of view. The PCA results indicated that the intraexperimental variations were well-controlled with no overlapping in the clusters of the three repeated hybridizations (red balls) of the each sample (Figure 4). The cluster analysis results indicated that the gene expression profile of the placebo-treated group, as compared to the baseline, showed greater similarity because there was overlapping in the two clusters (Figure 5). However, the two clusters are well separated in the DG-treated group. Therefore, the DG-treated group, has a greater change in the gene expression profile than the placebo-treated group. These results provide hints that DG might exert certain effects on postmenopausal subjects.

Two molecular pathways, (1) regulation of blood pressure (Figure 6) and (2) regulation of smooth muscle proliferation (Figure 7), were targeted by associating several hundreds of DEGs by using microarray and genome-wide association analysis. These two pathways were targeted only in the DG-treated group but not in the placebo-treated group. The functionally related genes EDN1, NOS3, PTGS2, ILK, ELANE, MFN2, ECE1, AVPR1B, DRD5, and ACE were either upregulated or downregulated in the DG-treated group and this resulted in negative regulation both in blood pressure and smooth muscle proliferation. 

 Our microarray data showed that DG treatment resulted in a lower level of EDN1 and a higher level of NOS3 as compared to the placebo treatment. Endothelin-1 (EDN1), a member of the endothelin family, has been known to be the most potent and long-acting vasoconstrictor with mitogenic activity [15, 16]. It is released mainly from the basal surface of the endothelium and it is partially synthesized in and released from smooth muscle cells (SMCs). It acts on vascular SMC in a paracrine and autocrine fashion by controlling the basal vascular tone [17]. Upregulation of EDN1 has been correlated with hypertension. Proliferation of SMC is a key feature of several pathogenic processes, including atherosclerosis and restenosis after coronary angioplasty. Because EDN1 also possesses mitogenic properties, it plays a role in regulating the proliferation of intimal smooth muscle cells and inducing IMT in atherosclerosis [18]. Endothelial NOS (eNOS), also known as nitric oxide synthase 3 (NOS3), is an enzyme that is encoded by the NOS3 gene. Nitric oxide (NO), a potent vasodilator constitutively produced by eNOS, is thought to be the endothelium-derived relaxing factor that mediates relaxation [19, 20] in blood vessels and is involved with regulating vascular tone by inhibiting the smooth muscle contraction which is an important element for the prevention of hypertension. NO, which was synthesized by eNOS, diffuses to underlying SMCs and stimulates one of its molecular targets, namely, soluble guanylate cyclase. Generated cGMP then mediates biological responses, including vasorelaxation, inhibition of cell proliferation and migration, and extracellular matrix production [21, 22]. Gene transfer studies have proved that adenoviral eNOS delivery to balloon-injured rat carotid arteries restores vascular NO production and reduces neointima formation, at least in part because of the antiproliferative effect on SMC [23, 24]. Therefore, a lower level of EDN1 and a higher level of NOS3 after DG treatment suggest an antihypertensive effect and reduction in neointima formation in these two key DEGs. 

 Endothelin-1 (ET-1) is formed by the cleavage of the endothelin precursor, big endothelin (big-ET), by an endothelin-converting enzyme (ECE1). Hence, the inhibition of ECE1 leads to a reduction of physiologically active ET-1 and the associated vasoconstricting activity of this molecule. One clinical study found that the ECE1 C-339A polymorphism was genotyped in 698 women in which 669 women could also be genotyped for EDN1 K198N polymorphism. The results from this large association suggest that the genes ECE1 and EDN1 interact to modulate blood pressure levels in women [25]. Our study also found that DG treatment could significantly downregulate both ECE1 and EDN1 in postmenopausal women. 

 Arginine vasopressin receptor 1B (AVPR1B, previously known as vasopressin 3 receptor or antidiuretic hormone receptor 1b) is a protein that acts as a receptor for arginine vasopressin (AVP). A study of the role of AVP for the development of hypertension after constriction of the abdominal aorta proximal to the renal arteries concluded that AVP plays an important role in the development of hypertension and that the action is mediated via the vascular AVP-receptor (AVPR1B) [26]. Angiotensin I-converting enzyme (ACE), an exopeptidase, is a circulating enzyme that participates in the body's rennin-angiotensin system, which mediates extracellular volume and arterial vasoconstriction. This enzyme plays a key role in the production of angiotensin II and in the catabolism of bradykinin, two peptides involved in the modulation of vascular tone and in the proliferation of SMC [27]. DRD5 gene encodes the D5 subtype of the dopamine receptor. The D5 receptor is important in blood pressure regulation. A study used D5 dopamine receptor knockout mice to try to correlate with hypertension, and it was found that disruption of the D5 gene increases blood pressure [28, 29]. Dopamine was found to interact with AVP, and the increase in blood pressure caused by AVP can be opposed by dopamine [30]. DRD5 upregulation could be beneficial to hypertension. Based on the downregulation of AVPR1B and ACE and the upregulation of DRD5, DG treatment may be involved in the rennin-angiotensin system and the receptor-mediated improvement of hypertension.

 The gene PTGS2 encodes prostaglandin-endoperoxide synthase (PTGS), also known as cyclooxygenase (COX-2), and it is the key enzyme in prostaglandin biosynthesis. It is responsible for the prostanoid biosynthesis which involves inflammation and mitogenesis [31]. The activation of this gene is an early response to injury in vascular SMC. In an *in vivo* experimental model of balloon angioplasty of the carotid artery, it was shown that the COX-2 protein is induced in arterial SMC of the injury. SMC activation in arterial vessels walls after balloon injury is prolonged, with rounds of migration and division of SMC lasting up to two weeks [32, 33]. Integrin-linked kinase (ILK) is a 59 kDa serine-threonine kinase which has been associated with multiple cellular functions, including cell migration, cell proliferation, cell-adhesions, and signal transduction. Following a balloon catheter injury of the rat carotid artery *in vivo*, a dramatic decrease in ILK levels coincided with both the proliferation and the migration of SMCs, which leads to the formation of a thickened neointima. An increase in the ILK level may inhibit SMC migration, proliferation, and neointimal thickening [34]. The gene ELANE encodes serine proteases elastases that degrade the extracellular matrix, releasing growth factors and chemotactic peptides, inducing glycoproteins such as tenascin, and thereby promoting vascular cell proliferation and migration. It was found that elastase activity was induced by carotid arterial injury in mice together with inflammatory cells adherent to or within the vessels [35]. The inhibition of elastase could be a therapeutic target for neointimal thickening. The MFN2 gene provides instructions for making a protein called mitofusin 2. This protein helps to determine the shape and structure (morphology) of the mitochondria, the energy-producing centers within cells. In addition, MFN2 is an important suppressor of vascular SMC proliferation and it can trigger SMC apoptosis via a mitochondrial death pathway. Recent studies have demonstrated that MFN2 acts as an endogenous Ras inhibitor and that deregulation of MFN2 expression leads to vascular proliferative disorders in the settings of genetic hypertension, atherosclerosis, and restenosis after vascular injury. In addition, the overexpression of MFN2 overtly suppresses the mitogenic stimulus-evoked vascular SMC proliferation in culture, and it also blocks balloon injury-induced restenosis *in vivo* via inhibiting the Ras-Raf-MEK-ERK/MAPK signaling pathway [36]. Based on the downregulation of PTGS2, ILK, and ELANE and the upregulation of MFN2, DG treatment may involve the multiple negative regulation of neointimal thickening by inhibiting vascular SMC proliferation and migration in mitogen-activated protein kinase pathways and signal transducers (MAPK) and be an activator of the transcription protein pathway (JAK-STAT). Our results also showed the downregulation of co-citation genes, MAPK1, MAPK14, STAT3, and STAT5B, in the abovementioned pathways and this may imply that DG treatment may improve neointimal thickening via the inhibition of SMC proliferation. 

By integration of DEGs in these two biological processes and performing network analysis using GePS, both EDN1 and NOS3 were the core of the network (Figure 10). Endothelin-1 (EDN1) is a potent vasoconstrictor, it is overexpressed in the vasculature in hypertension, and it is able to increase vascular growth [37]. The endothelial nitric oxide synthase (NOS3) gene is positively associated with hypertension [38] and with inhibited vascular SMC proliferation and neointimal formation [23]. As a consequence, the downregulation of EDN1 and the upregulation of NOS3 could have antihypertension and inhibition of intimal smooth muscle proliferation [39]. 

 The clinical outcome of carotid IMT reduction after DG treatment in the same study showed consistent observation with this molecular target study with the mechanism elucidated. The other finding from molecular targeting is the antihypertensive effect of DG treatment, as shown in the patient's blood samples. However, there is no change in blood pressure of a patient in this clinical study, because the subjects are all in normotensive state. But our previous *in vivo* study showed the antihypertensive effect of DG in a spontaneous hypertensive rat [6]. Although no relationship can be found regarding the antihypertensive effect of DG between clinical and molecular studies, a molecular targeting study shows the power of revealing the hidden properties of DG treatment that could not be shown in the setting of a clinical study. 

## 5. Conclusions

 This genome-wide association study on the molecular action of DG in postmenopausal women with hypercholesterolemia using 18 treatments and 11 placebo samples before and after treatment has gathered sufficient molecular targets and pathways to reveal that herbal formula DG could improve neointima thickening and hypertension. 

## Supplementary Material

Gene Expression Profiling on the Molecular Action of Danshen-Gegen Formula in a Randomized Placebo-Controlled Trial of Postmenopausal Women with Hypercholesterolemia

## Figures and Tables

**Table 1:** RT-PCR primer pairs for the gene identified in the microarray analysis.

	Primer sequence (5′→3′)	
	Forward	Reverse
ACE	TGGTGACTGATGAGGCTGAG	TCTTGCTGGTCTCTGTGGTG
AVPR1B	CCTGGCTATCTTCGTTCTGC	CCAGGCCTGTGTCTTGACTT
CTSB	TTGCCAACTCCTGGAACACTGACT	AGGCCCACGGCAGATTAGATCTTT
DRD5	TCATCTATGCCTTCAACGCCGACT	ATGTAGGCAGCTGCGATTTCCTTG
ECE1	ACCTGTCTTCCTCGCTGGAAGTTT	TCGGGTTTCCTCATCCATCCACTT
EDN1	GGGGATCTGAGTCTGTCCAA	CAACACACATGCTGGGAAAC
ELANE	CTGGGAGCCCATAACCTCTC	CACGATGTCGTTGAGCAAGT
ILK	GAGCCAGGCTGTGAAGTTTGCTTT	TCGGGCAGTCATGTCCTCATCAAT
MAPK1	ACGTTGGTACAGGGCTCCAGAAAT	TTCCCTGGAAAGATGGGCCTGTTA
MAPK14	AGTCCTGAGCACCTGGTTTCTGTT	ACATGCACACACACTAACACGCAC
PTGS2	CAAATCCTTGCTGTTCCCACCCAT	GTGCACTGTGTTTGGAGTGGGTTT
MFN2	AGCCCTGGTATTGATGTCACCACA	ATGAAGATGTTTGGCCGGGAGAGA
NOS3	CCCTTCAGTGGCTGGTACAT	TATCCAGGTCCATGCAGACA
NPPA	GGGTCTCTGCTGCATTTGTGTCAT	AGAGGCGAGGAAGTCACCATCAAA
NR3C1	TGAAGGTTTCTGCGTCTTCACCCT	CTGCGCATTGCTTACTGAGCCTTT
PRKCB	ACCGCCTGTACTTTGTGATGGAGT	CCGATGGCAATTTCTGCAGCGTAA
PRKCD	TGCCGCTGAGATAATGTGTGGACT	TTGCACATCCCAAAGTCGGCAATC
RAF1	TGCCGAACAAGCAAAGAACAGTGG	AGTCTGAACACTGCACAGCACTCT
STAT3	ATGGAAGAATCCAACAACGGCAGC	AGGTCAATCTTGAGGCCTTGGTGA
STAT5B	AAATTCAAGGCCGAAGTGCAGAGC	CATCACACCGTCAAACCATTGCCA
GAPDH	ACAGTCAGCCGCATCTTCTT	ACGACCAAATCCGTTGACTC

**Table 2:** Patient baseline characteristics.

Item	DG (*n* = 18)	Placebo (*n* = 11)	*P* value
Age (year)	56.7 ± 4.8	56.1 ± 3.8	0.663
Minimal	47.0	48.0	
Maximal	65.0	63.0	
Body weight (kg)	53.8 ± 5.4	54.3 ± 7.4	0.779
BMI (kg/m^2^)	22.3 ± 2.3	23.0 ± 2.7	0.400
SBP (mmHg)	125.3 ± 14.5	127.3 ± 10.5	0.635
DBP (mmHg)	72.6 ± 11.7	74.7 ± 8.7	0.525
TC (mmol/L)	6.6 ± 0.6	6.3 ± 0.5	0.091
LDL (mmol/L)	4.3 ± 0.5	4.0 ± 0.4	0.052
HDL (mmol/L)	1.8 ± 0.4	1.7 ± 0.3	0.258
TG (mmol/L)	1.1 ± 0.4	1.4 ± 0.8	0.229
Glucose (mmol/L)	5.1 ± 0.4	5.0 ± 0.4	0.483
Creatinine (umol/L)	64.9 ± 7.5	61.3 ± 6.1	0.106
Urea (mmol/L)	5.2 ± 1.0	4.7 ± 0.9	0.117
IMT (mm)	0.784 ± 0.125	0.763 ± 0.101	0.563
Plaque			
Yes	9	5	0.273
No	12	14	
Plaque index	1.56 ± 0.88	2.00 ± 1.73	0.530

**Table 3:** Blood pressure of subjects at baseline and 12 months after DG or placebo treatment.

Item	Systolic BP (mmHg)		Diastolic BP (mmHg)	
	Baseline	12 months	Baseline	12 months
TCM (*n* = 18)	125.3 ± 14.5	120.0 ± 16.8	72.6 ± 11.7	74.9 ± 10.8
Placebo (*n* = 11)	127.3 ± 10.5	123.8 ± 9.8	74.7 ± 8.7	76.7 ± 8.9
*P* value^#^	0.635	0.552	0.525	0.869

^#^Compared between groups (Mann-Whitney test).

**Table 4:** Genes regulated after DG treatment in microarray analysis. Blood pressure-related genes are shown in identified genes which are shown in orange color while co-citation genes are shown in grey color.

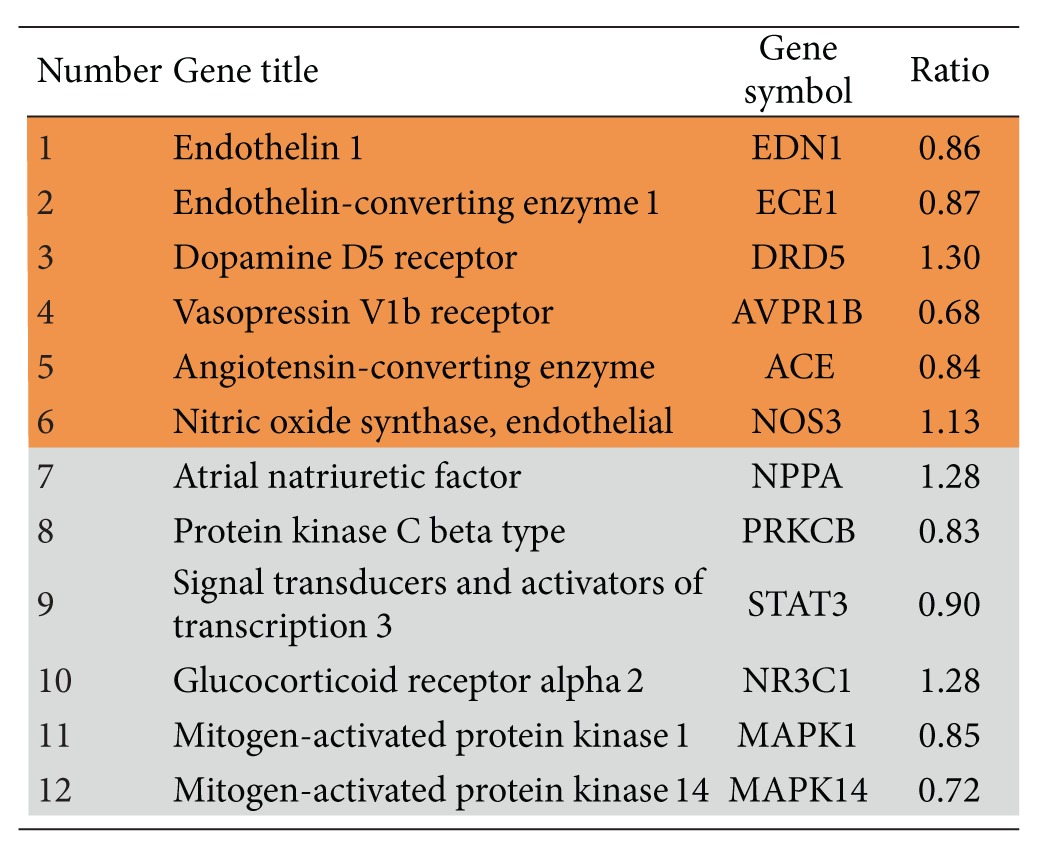

**Table 5:** Genes regulated after DG treatment in microarray analysis. Smooth muscle proliferation-related genes are shown in identified genes which are shown in orange color while co-citation genes are shown in grey color.