# Supplementary material for: Gene Expression Profiling on the Molecular Action of Danshen-Gegen Formula in a Randomized Placebo-Controlled Trial of Postmenopausal Women with Hypercholesterolemia
Source: Evid Based Complement Alternat Med. 2013 Sep 23;2013:703705. doi: 10.1155/2013/703705 (PMC3794622; doi:10.1155/2013/703705)
Supplement: Supplementary file 1 — Gene Expression Profiling on the Molecular Action of Danshen-Gegen Formula in a Randomized Placebo-Controlled Trial of Postmenopausal Women with Hypercholesterolemia [file 703705.f1.pdf]

Supplementary: Inclusion and exclusion criteria of the clinical study

Eligibility criteria for participants

1. 45-65 year of age
2. menopause for more than 12 months
3. fasting serum LDL ≧3.5 mmol/L
4. not taking hormones, statins or any nutritional supplements
5. No drug use
6. No calcium supplement
7. Have children(s)
8. Non-vegetarian

Exclusion Criteria

1. have severe medical diseases
2. fasting blood sugar ＞ 7.0 mmol/L
3. sitting blood pressure＞140/90mmHg
4. creatinine＞100 μmol/L
5. triglyceride ＞11.3mmol/L
6. angina symptoms or with significant ischaemic ECG changes
7. current smoking habit
8. using the lipid lowering drugs within the past 4 weeks
9. taking warfarin currently
10. history of herb hypersensitivity
